# Exploring temporal impact of important factors on cardiac events prediction in heart failure using a random survival forest model

**DOI:** 10.1093/ehjopen/oeaf107

**Published:** 2025-08-18

**Authors:** Daisuke Harada, Takahisa Noto, Junya Takagawa, Kazuaki Fukahara

**Affiliations:** The Cardiology Division, Imizu Municipal Hospital, 20 Hohnoki, Imizu City, Toyama 934-0053, Japan; The Cardiology Division, Imizu Municipal Hospital, 20 Hohnoki, Imizu City, Toyama 934-0053, Japan; The Cardiology Division, Imizu Municipal Hospital, 20 Hohnoki, Imizu City, Toyama 934-0053, Japan; The Cardiology Division, Imizu Municipal Hospital, 20 Hohnoki, Imizu City, Toyama 934-0053, Japan

Improvements in the outcomes of heart failure (HF) require timely, individualized treatment and the prevention of cardiac events. Machine learning enables more accurate predictions than traditional methods.^1–3^ Random survival forest (RSF), a machine learning algorithm, captures complex non-linear relationships and builds flexible, high-performance models for predicting cardiac events.^4^ It also identifies important features associated with these predictions. This study aimed to develop a high-performance RSF model that predicts cardiac events in HF patients and examine how the importance of predictors changes over time.

This study was approved by our institutional review board (ethics approval number: 024007). We retrospectively analyzed echocardiographic, jugular venous pulse, and medical record data from April 2017 to September 2021, initially identifying 946 patients. Inclusion criteria followed our previous study.^5^ HF was defined as brain natriuretic peptide (BNP) ≥ 40 pg/mL with HF symptoms/signs or a history of HF hospitalization, regardless of left ventricular ejection fraction (LVEF).^6^ We excluded patients with specific cardiac diseases, incomplete data, no HF symptoms, or no follow-up at our hospital. As a result, 381 symptomatic HF patients were included. *Figure 1A* shows the RSF model flowchart. Data were split into validation and test sets (80:20). We used 5-fold cross-validation and hold-out methods to assess performance and overfitting. The RSF model was built using 42 clinical features (*Figure 1B*). Performance was evaluated using concordance index, integrated Brier score (BS), integrated cumulative/dynamic area under curve (C/D AUC), and time-series changes in BS and C/D AUC. BS loss after permutations subtracted by loss in the full model was also used to identify important features for predicting cardiac events.^7^ Cardiac events were defined as a composite of sudden death, HF death, emergent loop diuretic use, or HF hospitalization.

**Figure 1:**
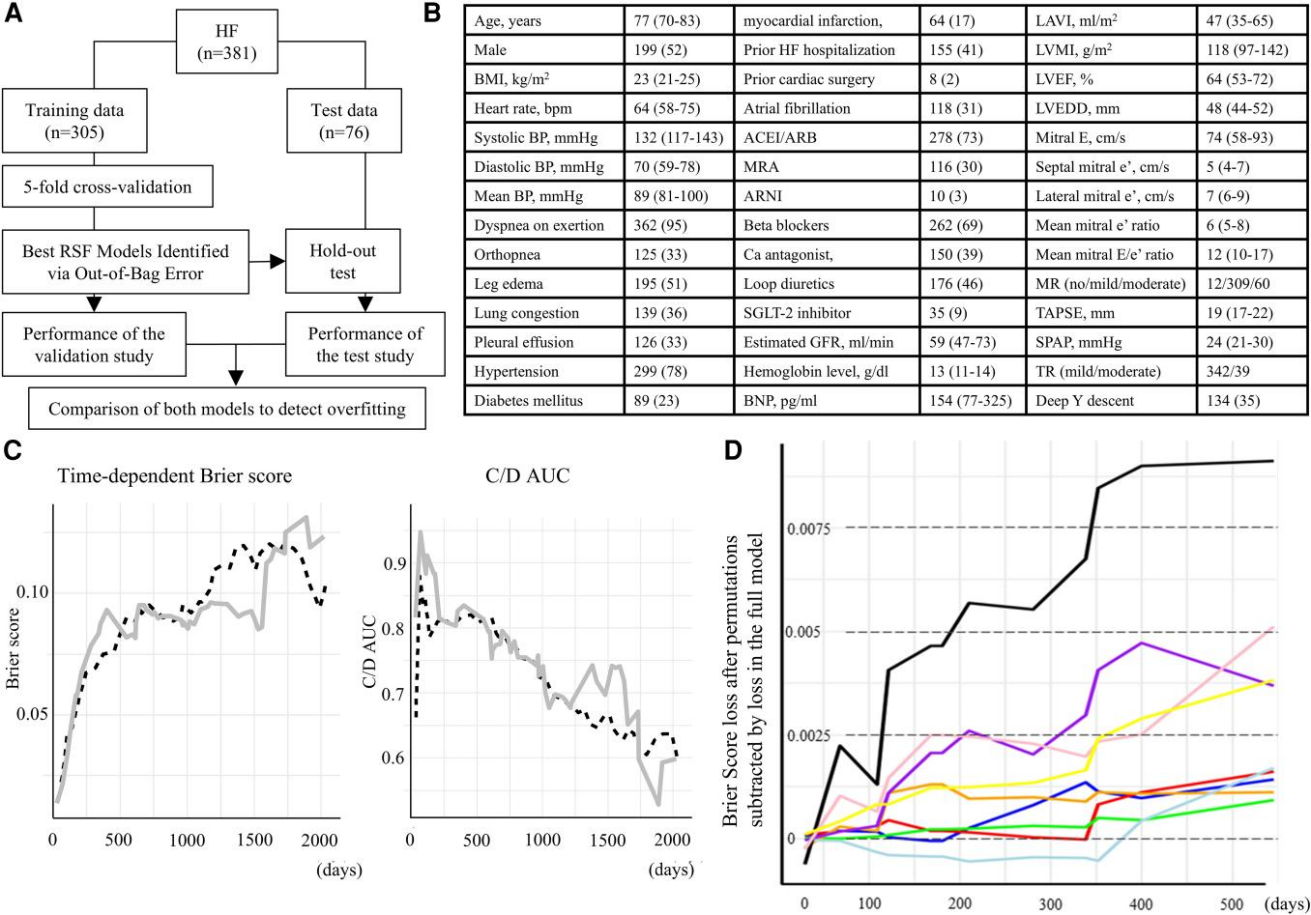
(*A*) Study flow chart. (*B*) Clinical feature. Data are shown as the number of patients (%), or a median (interquartile range). (*C*) Time-dependent changes in the average Brier score (left) and cumulative/dynamic AUC (right); dashed black line, validation; gray line, test. (*D*) Average Brier score loss after permutation minus that of the full model, up to ∼540 days. Coloured lines: black, BNP; pink, estimated GFR; yellow, hemoglobin; purple, loop diuretic use; orange, LVEF; blue, deep Y descent; red, mean mitral E/e′; light blue, SPAP; green, TAPSE. ACEI/ARB, angiotensin-converting enzyme inhibitors/angiotensin receptor blockers; ARNI, angiotensin receptor-neprilysin inhibitor; BMI, body mass index; BNP, brain natriuretic peptide; BP, blood pressure; C/D AUC, cumulative/dynamic area under the curve; GFR, glomerular filtration rate; HF, heart failure; LAVI, left atrial volume index; LVEDD, left ventricular end diastolic dimension; LVEF, left ventricular ejection fraction; LVMI, left ventricular mass index; MR, mitral regurgitation; MRA, mineralocorticoid receptor antagonist; RSF, random survival forest; SGLT-2, sodium glucose co-transporter 2; SPAP, systolic pulmonary arterial pressure; TAPSE, tricuspid annular plane systolic excursion; TR, tricuspid regurgitation.

The mean follow-up time was 1009 ± 576 days, during which 90 cardiac events were observed. The concordance index, integrated BS, and integrated C/D AUC for the validation and test models were similar (medians: 0.871 vs. 0.894, 0.102 vs. 0.093, and 0.713 vs. 0.730, respectively). Temporal trends in average BS and C/D AUC were also comparable (*Figure 1C*), suggesting minimal overfitting. The models performed well, with C/D AUC >0.8 from the time of cardiac function assessment up to 540 days thereafter. As no absolute threshold for BS loss is established, we used relative ranking to identify key predictors. *Figure 1D* shows temporal changes in the top nine variables with the greatest BS loss over the 540-day period: BNP, the estimated glomerular filtration rate, hemoglobin, the use of loop diuretics, LVEF, a deep Y descent, the mean mitral E/e′ ratio, systolic pulmonary arterial pressure, and tricuspid annular plane systolic excursion.

To the best of our knowledge, this is the first study to show the time-dependent predictive relevance of risk factors for cardiac events, including right ventricular function, using high-performance RSF models.

Based on these results, patients with elevated BNP levels, a well-known prognostic marker in HF,^8^ need to be scheduled for early follow-up visits due to their increased risk of cardiac events in the early phase. Patients with normal BNP levels, preserved renal function, and adequate hemoglobin levels may benefit from extended follow-up intervals, potentially reducing unnecessary medical costs without compromising safety. Reduced LVEF and impaired right ventricular function are both significant risk factors for cardiac events.^9,10^ However, these cardiac dysfunctions differ in their temporal association with cardiac events. Our results show that reduced LVEF is more closely associated with an earlier onset of cardiac events than impaired RV function, suggesting that LV dysfunction warrants earlier intervention. The RSF model’s ability to capture time-dependent risk allows clinicians to align the timing and intensity of follow-ups with each patient’s evolving risk profile. When treatment strategies are modified according to these predictors, the periodic reassessment of event incidence is essential to confirm their effectiveness. The present results support a dynamic, patient-tailored approach to HF management, balancing clinical effectiveness and resource efficiency. Therefore, beyond predictions, our RSF model may play a key role in supporting personalized HF management. There are several limitations that need to be addressed. Although our RSF model used 42 variables, a simplified model, such as that using the top nine predictors, may be more interpretable and feasible for clinical use. Machine learning methods such as RSF show promise for risk prediction, but typically require large cohorts to ensure stable results. In this study, the sample size decreased from 946 to 381, which may have introduced a selection bias. Although validation and test performances were similar, suggesting limited impact from data dependency and random variations, these inherent biases remain important limitations that may have affected the generalizability of our results. Therefore, larger and more diverse cohorts are needed to confirm and extend these results. Factors related to cardiac events do not necessarily become risk factors for the onset of cardiac events at the same time. Our model may provide personalized medical care suited to timing based on the probability of cardiac event occurrence.

## Data Availability

The data underlying this article will be shared on reasonable request to the corresponding author.

## Lead author biography

Dr Daisuke Harada is a cardiologist from Japan. After completing his training at the University of Toyama, he worked as a cardiology fellow at Imizu Municipal Hospital in Japan. He is currently practicing as a clinician at the same hospital. His main research interests include elucidating the pathophysiology of heart failure, exploring the relationship between heart failure and sympathetic nerve activity, and the clinical application of artificial intelligence.
